# Absence of BBSome function leads to astrocyte reactivity in the brain

**DOI:** 10.1186/s13041-019-0466-z

**Published:** 2019-05-09

**Authors:** Minati Singh, Janelle E. Garrison, Kai Wang, Val C. Sheffield

**Affiliations:** 1 Department of Internal Medicine, University of Iowa, Iowa City, IA 52242 USA; 2 Departments of Pediatrics and Ophthalmology, University of Iowa, Iowa City, IA 52242 USA; 3 Department of Biostatistics, University of Iowa, Iowa City, IA 52242 USA

**Keywords:** BBS, Reactive astrocytes, Neuroinflammation, And microglia

## Abstract

In humans, dysfunctional primary cilia result in Bardet-Biedl syndrome (BBS), which presents with clinical features including intellectual disabilities, obesity, and retinal degeneration, and, in mouse models, the added feature of hydrocephalus. We observed increased Glial Fibrillary Acidic Protein (GFAP) immunoreactivity in BBS mouse brains. Increased GFAP expression is a hallmark of astrocyte reactivity that is associated with microglia activation and neuro-inflammation. To gain a better understanding of reactive astrocytes observed in BBS mice, we used two mouse models of BBS8, a BBSome protein, to characterize the reactive astrocyte phenotype. The finding of reactive astrocytes in young BBS mouse brains led us to hypothesize that loss of BBSome function leads to reactive astrocytes prior to hydrocephalus and obesity. By using two mouse models of BBS8, a congenital BBS8 knockout with hydrocephalus, and a tamoxifen-inducible BBS8 knockout without hydrocephalus, we were able to molecularly phenotype the reactive astrocytes. Molecular phenotype of reactive astrocytes shows differential regulation of inducers of Pan, A1 neurotoxic, and A2 neuroprotective astrocytes that are significantly altered in brains of both congenital and induced knockouts of BBS8, but without microglia activation. We find evidence for neuroinflammation in the brains of congenital knockout mice, but not in induced knockout mice. Protein levels of GFAP, SERPINA3N and post-synaptic density 95 (PSD95) are significantly increased in congenital knockout mice, but remain unchanged in induced knockout mice. Thus, despite the reactive astrocyte phenotype being present in both models, the molecular signature of reactive astrocytes in BBS8 mice models are distinct. Together, these findings suggest that BBS8, and by extension the BBSome, plays a role in neuro-astrocyte functions independent of hydrocephalus, and its dysregulation is associated with astrocyte reactivity without microglia activation. (Total word count 278).

## Introduction

Neuro-inflammation of the central nervous system (CNS) is marked by the presence of reactive astrocytes [1]. Astrocytes are the most numerous types of glial cell and have close associations with neurons [2, 3]. Astrocytes have many roles including maintaining the blood brain barrier, neurotransmission, synaptogenesis, metabolic regulation, and supporting synaptic transmission [4, 5]. Astrocytes also nourish neurons by maintaining and pruning neurons, clearing unused neurotransmitters, and providing nutrients to neurons [6].

Reactive astrocytes, which originate in response to an insult to the brain, are highly disease and context specific [7, 8]. This makes it difficult to assign a specific, unified molecular signature to reactive astrocytes. However, recent work has identified two different subtypes of reactive astrocytes: A1 and A2 [9]. Subtype A1 reactive astrocytes are neurotoxic and are induced by reactive microglia, which are the primary immune cell in the brain. A1 reactive astrocytes weaken synapses, disrupt synaptic signaling, and cause the death of neurons and myelin-producing oligodendrocytes. A1 reactive astrocytes have been observed in injuries and neurodegenerative diseases such as Alzheimer’s disease, Huntington’s disease, and Multiple Sclerosis [10–15]. Subtype A2 reactive astrocytes, on the other hand, tend to be neuroprotective. A2 astrocytes are supported by the microglia, which secrete cytokines that promote neuronal survival.

Primary cilia are antennae-like extensions of the cellular membrane that have diverse functions. Ciliary defects result in an array of clinical manifestations that are collectively known as ciliopathies [16, 17]. Bardet–Biedl syndrome (BBS) is an autosomal recessive, pleiotropic heterogeneous ciliopathy. Mutations in 21 different genes can independently result in BBS [18–22]. Of these, the proteins coded by *BBS1, BBS2, BBS4, BBS5, BBS7, BBS8, BBS9*, and *BBIP1/BBS18* interact to form a protein complex known as the BBSome. BBS6, BBS10, and BBS12 interact with TRiC chaperonin proteins to form a chaperone complex involved in assembling the BBSome. Other BBS proteins, such as BBS3 and BBS17, play a role in localization of the BBSome.

The characteristic phenotypes of BBS include, obesity, polydactyly, retinal degeneration, cardiac and renal malformations, brain abnormalities associated with hydrocephalus, and intellectual disability [23–25]. There is evidence to suggest that impaired ciliogenesis could lead to gliosis and neuro-inflammation. MRI brain scans from BBS patients show overall as well as region-specific changes in volume of the brain [25]. Additionally, primary cilia play a role in regulating inflammation [26, 27]. Furthermore, impaired ependymal ciliogenesis links neuroinflammation to hydrocephalus formation [28]. These data suggest that neuroinflammation could contribute to the neurological phenotypes observed in BBS patients.

We observed increased GFAP immunoreactivity and altered astrocyte morphology in the brains of 1-month old mice prior to obesity in congenital knockout mouse models of BBS1, BBS2, BBS4, and BBS8. This led us to test the hypothesis that loss of BBSome function leads to reactive astrocytes independent of hydrocephalus and to further characterize the molecular phenotypes of reactive astrocytes, reactive microglia, and neuro-inflammatory profiles of BBS mouse models utilized in this study. To assess the effects of loss of BBSome function on astrocyte reactivity and CNS inflammation, we examined the brains of BBS8 mutant mice at 1 month of age when the obesity phenotype is absent in BBS8 mice. We utilized inducible BBS8 mice as hydrocephalus is not present at 1 month of age [29] and this model allowed us to determine whether or not reactive astrocytes are secondary to hydrocephalus. We then generated molecular signatures of the reactive astrocytes from the brains of BBS8 congenital knockouts and BBS8 inducible knockout mice to determine whether molecular inducers of reactive astrocytes were present in the absence of hydrocephalus, and if so, what were the molecular phenotypes of reactive astrocytes. We used a subset of genes that have been reported to correspond to different types of reactive astrocytes and genes that impact astrocyte function by reactive astrocytes [9]. Since reactive astrocytes are associated with reactive microglia, neuroinflammation, and altered synaptic function, we examined whether reactive astrocytes observed in BBS8 mice were also associated with their molecular phenotypes of reactive astrocytes. Furthermore, numerous studies implicate a direct role for the BBSome in ciliary signaling by trafficking proteins in and out of cilia [30, 31]. We therefore examined several pre-and post-synaptic proteins and BBSome proteins to see if they are altered in the synaptosomal lysates. These experiments have allowed us to determine that the presence of reactive astrocytes occurs due to loss of BBSome function and is independent of hydrocephalus and microglia activation.

## Materials and methods

### Animal care and mice

The BBS mouse models utilized in this study have been described and characterized elsewhere [18–22, 29]. For the purposes of this manuscript, we have utilized *Bbs8*^*+/+*^ mice (hereafter referred to as WT), congenital BBS8 knockout mice (referred to as *Bbs8*^*−/−*^), *Bbs8*^*flox/flox*^; *Cre-* or *Bbs8*^*flox/−*^*; Cre-* mice (*Bbs8*^*flox*^; Cre-) and *Bbs8*^*flox/flox*^; *Cre +* or *Bbs8*^*flox/−*^*; Cre +* (*Bbs8*^*flox*^; *Cre+)* mice. *The Tamoxifen-Cre inducer mice were from Jackson Lab* UBC-Cre-ERT2 stock number 008085. All mice were housed in the animal facility of the University of Iowa. Experiments were approved by the Animal Care and Use Committee at the University of Iowa and conducted in accordance with the National Institutes of Health Guidelines for the Care and Use of Laboratory Animals. Both sexes of mice at 1 month of age were used in this study.

For *Bbs8*^*flox*^; Cre- and *Bbs8*^*flox*^; *Cre +* mice, 40 μl tamoxifen (15 mg/ml in corn oil) was injected subcutaneously at postnatal day 9, 12, and 15. Genotyping and excision efficiency was determined by PCR methodology as previously described [29]. We have observed no significant difference in excision efficiency between *Bbs8*^*flox/flox*^; *Cre +* and *Bbs8*^*flox/−*^*; Cre +* mice at this age [29]. At one month of age, *Bbs8*^*flox*^; Cre- and *Bbs8*^*flox*^; *Cre +* mice (2 weeks post tamoxifen), WT, and BBS8^-\-^ mice were cervically dislocated and brains excluding the cerebellum were harvested because increased GFAP immunoreactivity was not observed in this brain region of BBS8 mice. Brain tissue samples were snap frozen in liquid Nitrogen and stored at -80 °C for molecular analyses.

### Molecular studies

#### RNA expression

Total RNA was extracted from the whole brain minus cerebellum of cohorts of WT, *Bbs8*^*−/−*^, *Bbs8*^*flox*^; Cre-, and *Bbs8*^*flox*^; *Cre +* mice (*n* = 4/genotype) using an RNA isolation kit (Ambion Inc.). Following DNAse treatment, cDNA was generated from 500 ng of the DNAse-treated RNA using random hexamers. cDNA samples were diluted for use in the Power SYBRGreen assay (Applied Biosystem, Thermo Fisher Scientific). Primers used for analysis of pan-reactive and cytokine molecular markers (TaqMan assay, Table 1), A1 and A2 subtypes as identified by Liddelow and colleagues [9], and microglial expression (Table 2), were analyzed on a Bio-Rad CFX96 qPCR system.

**Table 1:** Taqman probes used in gene expression analyses of molecular inducers of pan reactive astrocytes, complement C3, and pro-inflammatory cytokines

Genes	Taqman Assay ID
*Gfap*	Mm01253033_m1
*Serpina3n*	Mm00776439_m1
*Vimentin*	Mm01333430_m1
*lcn2*	Mm01324470_m1
*Il15*	Mm00434210_m1
*Il16*	Mm0446190_m1
*Il1β*	Mm00434228_m1
*Gapdh*	Mm99999915_g1
*c3*	Mm01232779_m1

**Table 2:** Primers used for gene expression analysis of genes associated with microglia activation

Genes	Primers
*Iba1*	Forward: 5’gcagcacttgggtaacacct3’ Reverse: 5’taaccacccctcctttcctc
*Cd68*	Forward: 5’actggtgtagcctagctggt3’ Reverse: 5’ccttgggctataagcggtcc3’
*Tlr3*	Forward: 5’ccagaagaatctaatcaaattagatttgtc3’ Reverse: 5’ttttgctaagagcagttcttggag3’
*Tlr4*	Forward:5’ggcaacttggacctgaggag3’ Reverse:5’catgggctctcggtccatag3’
*Gapdh*	Forward: 5’catttcctggtatgacaatgaatacg3’ Reverse:5’tccagggtttcttactccttgga3’
*TMEM119*	Forward:5’gcatgaagaaggcctggac3’ Reverse:5’ctgggtagcagccagaatgt3’

#### Statistical analysis

Relative expression for each gene was normalized to GAPDH. The CT (cycle threshold) difference between GAPDH and the gene of interest (^Δ^CT) was determined. Mean differences were compared between *Bbs8*^*−/−*^ and WT littermates or *Bbs8*^*flox*^; Cre- and *Bbs8*^*flox*^; *Cre +* mice by using two sample unpaired student’s t-tests with 95% confidence to assess relative gene expression. The student’s *t*-test used to evaluate the significance of the differences of ^Δ^CT values (^ΔΔ^CT) between mutants and their corresponding controls (*n* = 4 per genotype). Log transformed relative mRNA expression levels are reported as mean ± standard error of the mean (SEM).

#### Protein extraction and western blot analysis

Total brain protein lysate was isolated from whole brain excluding the cerebellum (*n* > 4/genotype) using RIPA buffer (Cell signaling Inc.) and protease inhibitors. 20 μg of total brain protein lysate was subjected to western blot analyses and probed with primary antibodies at their respective dilution (Table 3) and their corresponding IRdyes secondary antibodies (Li-COR Biosciences). Protein bands were visualized by using the Odyssey Imaging system (Li-Cor Biosciences) and band densities were quantified using Image Studio software (Li-Cor Biosciences).

**Table 3:** Antibodies used either in western blot analysis or immunofluorescence imaging

Antibody	Company	Dilution
GFAP	Abcam	1:5000 western and immunofluorescence
SERPINA3N	R&D	1:2000 Western blot
LCN2	Abcam	1:2000 Western blot
SNAP25	Proteintech	1:3000 Western blot
CD68	Abcam	1:1000 Western blot
COMPLEXIN 2	Proteintech	1:2000 Western blot
PSD95	Santa Cruz	1:500 Western blot
IBA1	Abcam	1:1000 western blot and 1:200 Immunofluorescence
TTC8	Sigma	1:500 Western blot
GAPDH	Thermofisher	1:30000 western blot
HOMER	Santa Cruz	1:500 Western blot
GLUR1	Santa Cruz	1:1000 western blot
VIMENTIN	Abcam	1:1000 western and immunofluorescence
BBS2	Santa Cruz	1:200 western blot
BBS3	Proteintech	1:500 western blot
SYP	Proteintech	1:1000 western blot
NEUN	Abcam	1:1000 western blot and immunofluorescence
STBPX1	Proteintech	1:1000 western blot
VDAC	Abcam	1:1000 western blot
IL1β	Abcam	1:100 Immunofluorescence

#### Synaptosomal protein lysate isolation

Using a Dounce tissue grinder, the whole brain excluding the cerebellum was homogenized in 10 volumes of Syn-PER protein extraction reagent with protease inhibitor (Thermo Fisher Scientific Inc., #87793 and #87785, respectively) by applying 10 up-and-down even strokes. The homogenate was then centrifuged at 1200 x *g* for 10 min to remove cell debris. The supernatant was further centrifuged at 15,000×g for 20 min to obtain synaptosomal pellets. After centrifugation, synaptosomal pellets were gently solubilized and re-suspended in RIPA buffer with protease inhibitors for western blot analyses. 20 μg of total synaptosomal lysate was subjected to western blot analyses using corresponding primary and secondary antibodies (Table 3).

#### Statistical analysis

For Western blot analyses, the relative band density of each protein band was calculated by dividing the band density value of each protein by the band density of the corresponding loading control, GAPDH. After normalizing to GAPDH, using two sample unpaired student’s t-tests two samples were compared with 95% confidence to assess relative density differences between WT and *Bbs8*^*−/−*^ or *Bbs8*^*flox*^; Cre- and *Bbs8*^*flox*^; *Cre +* mice. Data are presented as relative mean signal intensity (arbitrary numbers) ± SEM and *p* < 0.05 was considered statistically significant in all tests.

### Immunofluorescence

Mice (*n* = 3/genotype) were perfused with 4% PFA and brains were removed and cryo-protected in 30% sucrose for 48 h. Following cryo-protection, 10 μm brain sections were mounted on Superfrost glass slides using the Leica CM30505 cryostat. Brain sections were washed in PBS 3 times for 5 min and blocked in PBS containing 0.1% triton X-100, 1% BSA for 1 h. After blocking, slides were incubated with primary antibodies (Table 3) overnight at 4 °C. The next day, slides were washed 3 times in PBS for 5 min followed by incubation in the corresponding Alexa Fluor secondary antibody for 1 h. Slides were then washed in PBS 3 times for 5 min and subsequently counter stained for Nuclei with VectaShield containing DAPI (Vector laboratories). Brain sections were matched as closely as possible to their corresponding wildtypes and images were captured with same parameters using confocal microscopy to represent immunoreactivity of various molecular markers associated with reactive astrocytes (Zeiss 710 confocal). For quantifying GFAP and VIMENTIN immunoreactivity from SVZ sub-regions of BBS8 mice (*n* = 4\genotype), NIH Image J software was used to assess the mean fluorescence intensity of GFAP and VIMENTIN.

## Results

### Congenital absence of BBSome function results in increased GFAP immunoreactivity

**Fig. 1:**
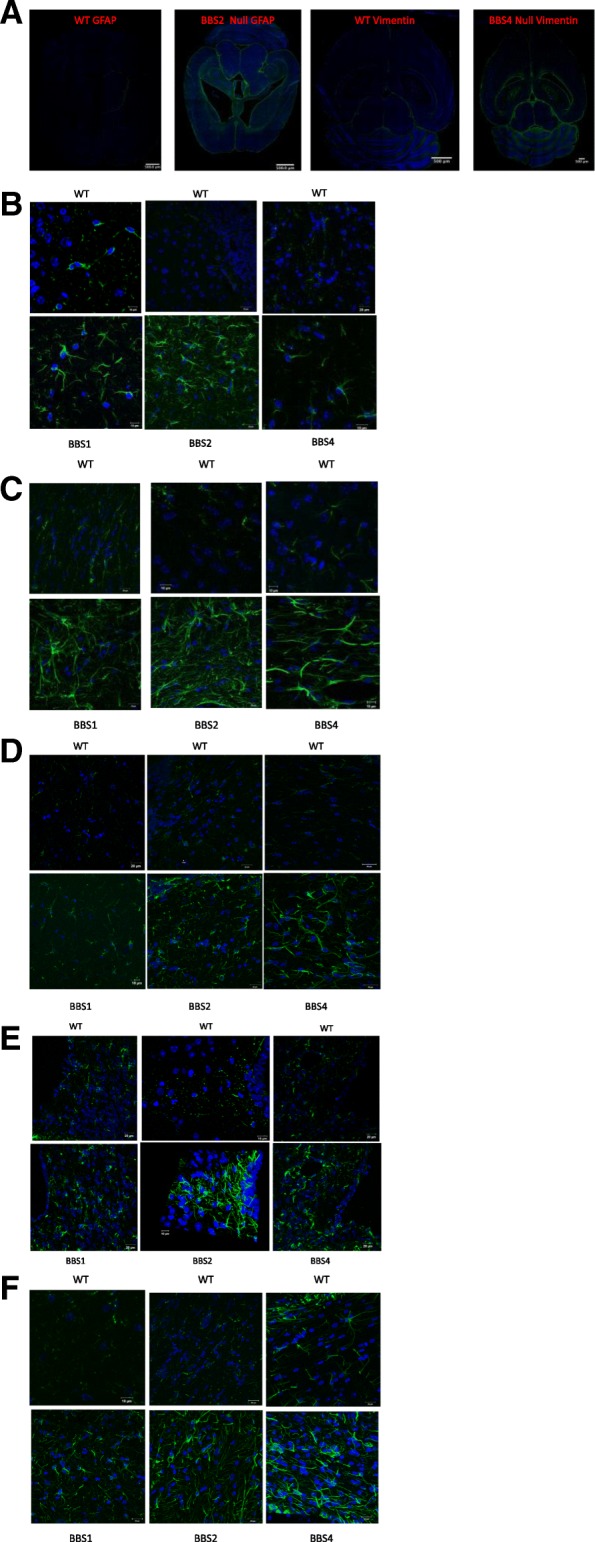
Congenital absence of BBSome function is associated with increased immunoreactivity in the brains of BBS mice as determined by GFAP, a pan reactive astrocyte marker. **a** Representative confocal 10X Tile images showing increased GFAP and VIMENTIN immunoreactivity in brains of BBS mice. Images were scanned using the same parameters for corresponding wildtype littermates (WT) and BBS null mice. **b-e** Best matched confocal images (63X magnification) of representative sub-brain regions from different strains of BBS mice showing increased GFAP immunoreactivity, a marker for reactive astrocytes, when compared to their WT littermates. Notably, increased GFAP immunoreactivity is observed in the hippocampus (**b**), corpus callosum(**c**), striatum(**d**), hypothalamus (**e**), and sub-ventricular zone (**f**) brain regions in BBS null mice when compared to their wild type littermates

**Fig. 2:**
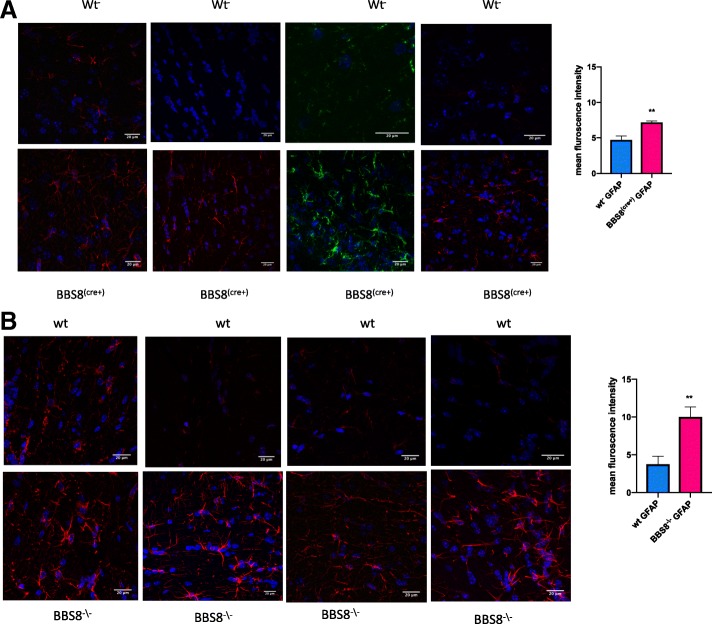
Loss of or reduced BBS8 protein levels is associated with significantly increased GFAP immunoreactivity (**a** & **b**) in sub-ventricular brain regions of BBS8 mice. Representative confocal images scanned with the same parameters at 63X magnification showing GFAP immunoreactivity (either green or red immunostained) in brains of corresponding control mice, *Bbs8*^*flox*^; *Cre +* mice, and *Bbs8*^*−/−*^ mice

**Fig. 3:**
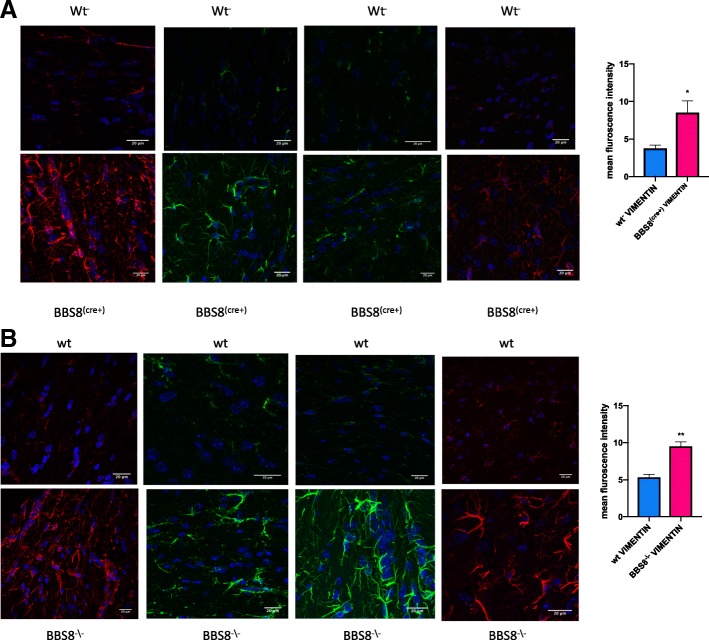
Loss of or reduced BBS8 protein levels is associated with significantly increased VIMENTIN immunoreactivity(**a** & **b**) in sub-ventricular brain regions of BBS8 mice. Representative confocal images scanned with the same parameters at 63X magnification showing VIMENTIN immunoreactivity (either green or red immunostained) in brains of corresponding control mice, *Bbs8*^*flox*^; *Cre +* mice, and *Bbs8*^*−/−*^ mice

In order to determine whether loss of BBSome function results in astrocyte activation, we initially assessed qualitatively the GFAP immunoreactivity in the brains of congenital knockouts for BBS1, BBS2, BBS4, and BBS8, as well as their respective wild type littermates using confocal microscopy (Fig. 1). We observe GFAP staining is more conspicuous in the white matter than in the gray matter in both control and knockout mice. What was common amongst several BBS models was that there is more intense GFAP immunoreactivity and that astrocytes have thicker and more numerous processes in BBS null mice (Fig. 1a, b-e). Amongst the genotypes of BBS mice, the increased GFAP immunoreactivity was particularly noticeable and common in the brain regions of corpus callosum, subventricular zone (SVZ) of the lateral ventricle (LV) adjoining the hippocampus, the striatum, the cortex, and the hypothalamus (Fig. 1b-e). Due to their heterogeneity, there is no single stereotypic event that can be assigned to reactive astrocytes. We report here the qualitative observed morphological differences in reactive astrocytes in best matched brain regions of BBS null mice to that of wildtype litter mate mice. However, in this study we used western blot analyses to quantify the protein expressions of various molecular markers associated with reactive astrocytes and microglia from whole brain lysates to gain a better understanding of molecular phenotypes of reactive astrocytes in BBS8 mice. In addition, we quantified the Immuno-stained brain sections obtained from wildtype littermates best matched SVZ brain sections from wild type and BBS8 mice and find there is significantly increased mean fluorescence intensity correlating with observed increased GFAP and VIMENTIN immunoreactivity in BBS8 mice (Fig. 2a and b and Fig. 3a & b; *n* = 4\genotype).

### Both congenital and postnatal loss of BBS8 function is associated with astrocyte reactivity, independent of hydrocephalus and obesity

Congenital knockout models of BBS have hydrocephalus, a phenotype often associated with reactive astrocytes. In order to determine whether the astrocyte reactivity in BBS is secondary to hydrocephalus and obesity, we utilized two different mouse models of BBS8, a component of the BBSome. The congenital *Bbs8*^*−/−*^ mice develop hydrocephalus in the late prenatal/early postnatal period and develop late onset obesity. We compared these mice to a mouse model of BBS8 in which the gene was deleted at P9–15 (*Bbs8*^*flox*^; *Cre+*) mice. *Bbs8*^*flox*^; *Cre +* mice do not have overt hydrocephalus and are not obese at one month of age.

**Fig. 4:**
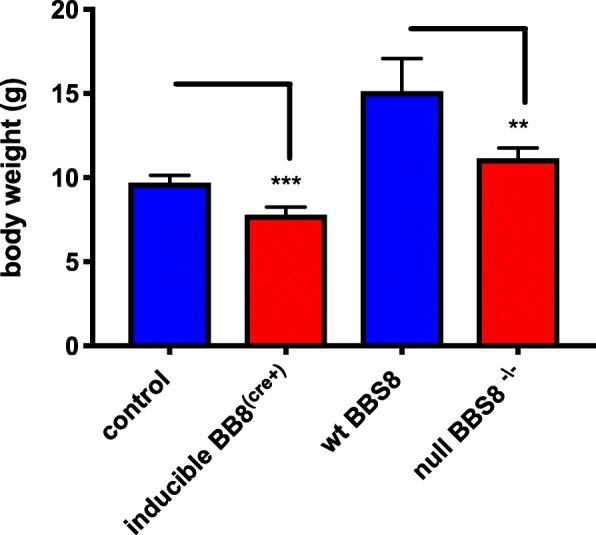
Significantly reduced body weights of mice with reduced or loss of BBS8 protein. Graphs shows mean + SEM body weights (g) of corresponding control mice, *Bbs8*^*flox*^; *Cre +* mice, and *Bbs8*^*−/−*^ mice at one months of age (*n* = 4\genotype, **p < 0.0023, ***p < 0.0001)

Since reactive astrocytes have been associated with the presence of obesity, a hallmark feature of BBS, we assessed whether our mutant mouse models were obese at 1 month of age. Both *Bbs8*^*−/−*^ (*p* < 0.0023) and *Bbs8*^*flox*^; *Cre +* (*p* < 0.0001) mice weigh significantly less than their corresponding control littermates (Fig. 4), making it unlikely that obesity contributes to the presence of reactive astrocytes at this time.

**Fig. 5:**
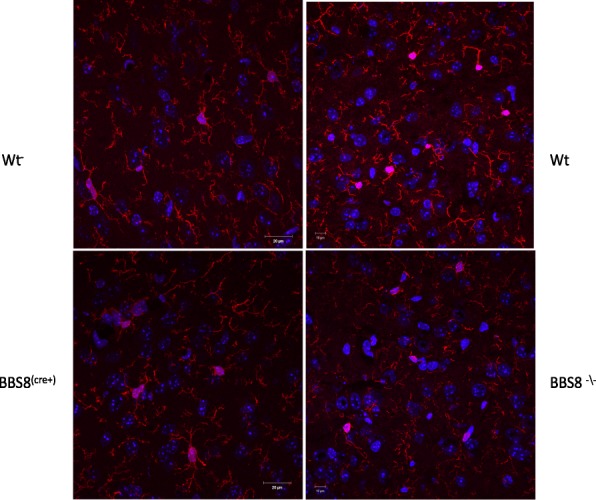
Loss of or reduced BBS8 protein levels is not associated with increased IBA1 immunoreactivity in cortex brain region of BBS8 mice. Representative confocal images scanned with the same parameters at 63X magnification showing IBA1 immunoreactivity in brains of corresponding control mice, *Bbs8*^*flox*^; *Cre +* mice, and *Bbs8*^*−/−*^ mice

**Fig. 6:**
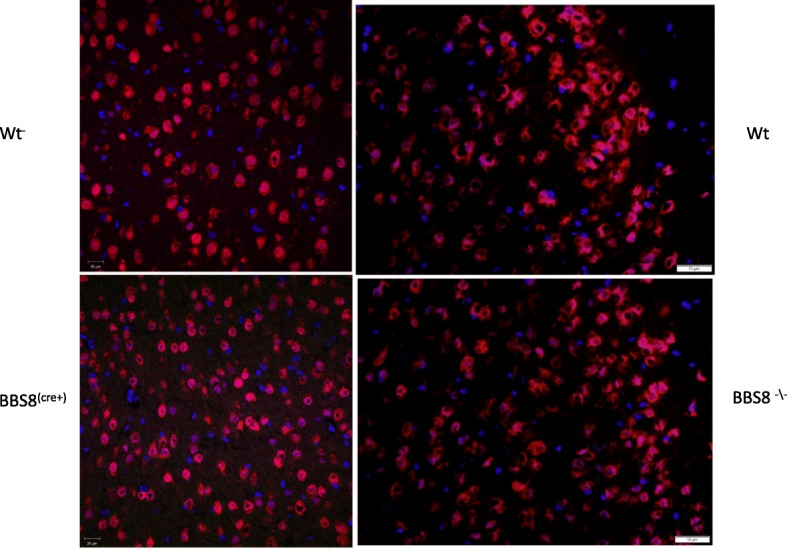
Loss of or reduced BBS8 protein levels is not associated with decreased NEUN immunoreactivity in cortex of BBS8 mice. Representative confocal images scanned with the same parameters at 40X magnification showing similar NEUN immunoreactivity in brains of corresponding control mice, *Bbs8*^*flox*^; *Cre +* mice, and *Bbs8*^*−/−*^ mice

At one month of age, we observed significantly increased GFAP immunoreactivity as measured by mean fluorescence intensity in both *Bbs8*^*−/−*^ and *Bbs8*^*flox*^; *Cre +* mice (Fig. 2a & b) compared to their respective control littermates. We also observed significantly increased immunoreactivity of VIMENTIN as measured by mean fluorescence intensity, another marker of reactive astrocytes, in mice lacking BBS8 function (Fig. 3a and b). We observed no differences in activated microglia, as stained by IBA1 (Fig. 5), or in neuronal cell number, as assessed by NeuN staining (Fig. 6). Together, these results indicate that loss of BBSome function leads to the presence of reactive astrocytes independent of hydrocephalus and does not cause either neuronal cell death or microglial activation at 1 month of age.

### Congenital absence of BBS8 causes activation of A1-like astrocytes, neuroinflammation, and altered postsynaptic density, independent of microglia activation

In order to corroborate the qualitative immunostaining results and we investigated the transcriptional changes associated with reactive astrocytes in congenital knockouts of BBS8, by performing gene expression analysis on total brain RNA from *Bbs8*^*−/−*^ and WT littermates (*n* = 4 for each genotype).

**Fig. 7:**
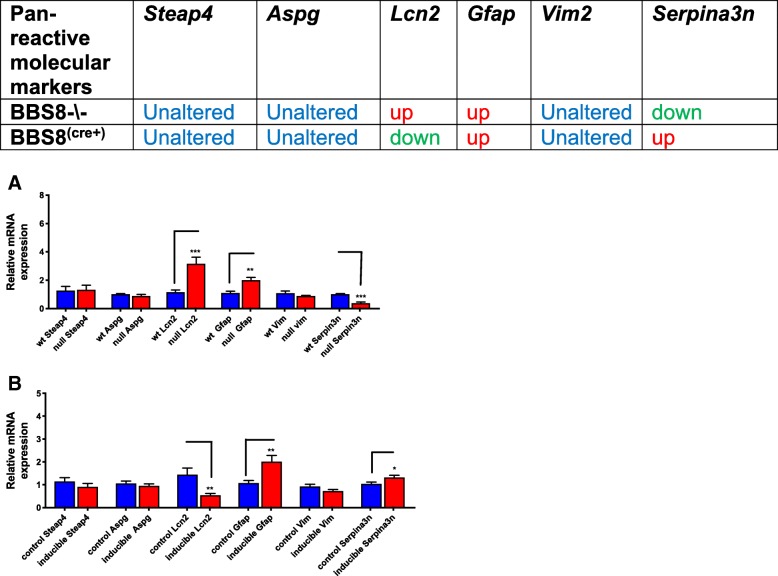
Loss or reduced BBS8 protein is associated with increased expression of pan-reactive molecular inducers of reactive astrocytes in brains of BBS8 mice. Expression of molecular inducers of pan-reactive astrocytes in brains of control mice, Cre+, and *Bbs8*^*−/−*^ mice are summarized and the graph shows relative mRNA expression of molecular inducers of Pan-reactive astrocytes in brains of control mice, Cre+, and *Bbs8*^*−/−*^ mice. Data are presented as Mean + SEM, **a**) wt and *Bbs8*^-\-^ mice (n = 4/genotype, **b**) *Bbs8*^*flox*^; *Cre-* and *Bbs8*^*flox*^; *Cre +* mice (*n* = 4/genotype, (n = 4/genotype, *P* value style APA: 0.12 non-significant, 0.033 (*, 0.002(**) and *p* < .001(***))

We observe increased expression of molecular markers of pan-reactive astrocytes *Gfap* (*p* < 0.001) and *Lcn2* (*P* < 0.001) in *Bbs8*^*−/−*^ mice, consistent with the presence of increased reactive astrocytes. However, *other known pan reactive molecular inducers; Serpina3n* expression (P < 0.001) is significantly reduced, and expression of *Steap4* (*p* > 0.05), *Aspg* (*p* > 0.05), and *Vimentin* (p > 0.05) are unaltered (Fig. 7a).

**Fig. 8:**
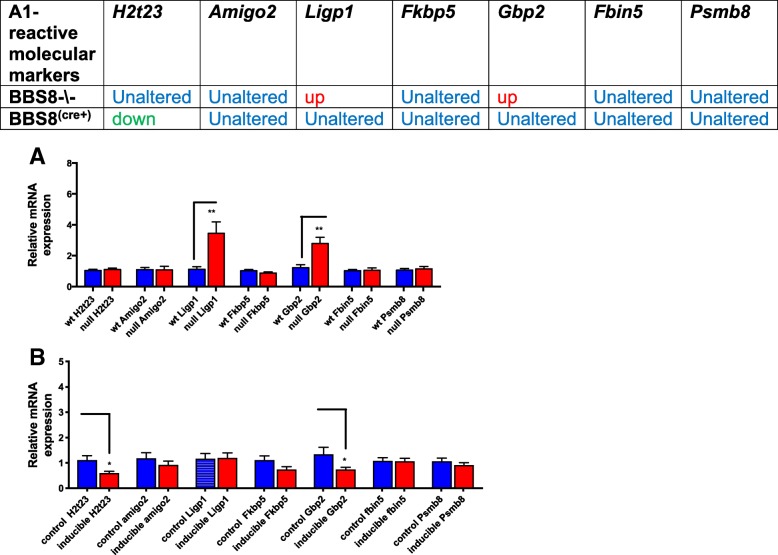
Loss of BBS8 protein is associated with increased expression of molecular inducers of A1 reactive astrocytes in brains of BBS8^-\-^ mice. Expression of molecular inducers of A1 reactive astrocytes in brains of control mice, Cre+, and *Bbs8*^*−/−*^ mice are summarized and graphed showing relative mRNA expression of molecular inducers of A1 astrocytes in brains of control mice, Cre+, and *Bbs8*^*−/−*^ mice. Data are presented as Mean + SEM. **a**) Wt and BBS8^-\-^ mice (n = 4/genotype, **b**) *Bbs8*^*flox*^; *Cre-* and *Bbs8*^*flox*^; *Cre +* mice (n = 4/genotype, P value style APA: 0.12 non-significant, 0.033 (*), 0.002(**) and *p* < .001(***))

**Fig. 9:**
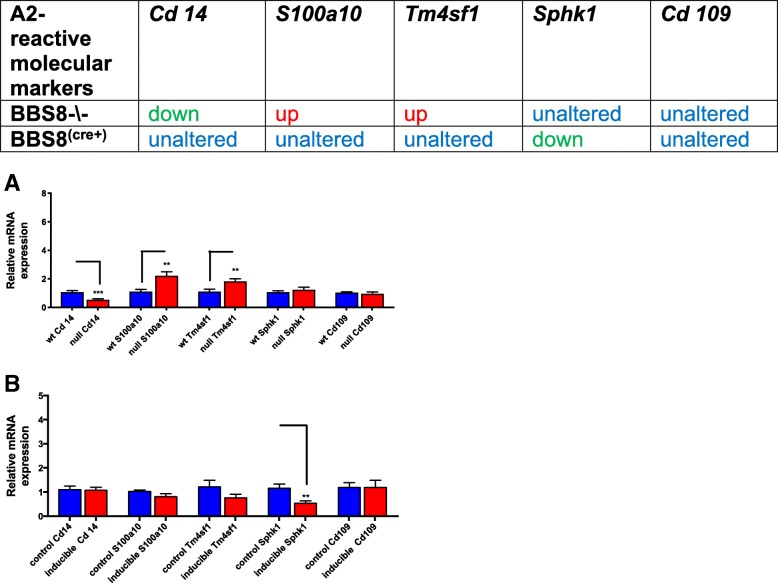
Loss of BBS8 protein is associated with increased expression of molecular inducers of A2 reactive astrocytes in brains of BBS8^-\-^ mice. Expression of molecular inducers of A2 reactive astrocytes in brains of control mice, Cre+, and *Bbs8*^*−/−*^ mice are summarized and graphed showing relative mRNA expression of molecular inducers of A2 astrocytes in brains of control mice, Cre+, and *Bbs8*^*−/−*^ mice. Data are presented as Mean + SEM. **a**) wt and BBS8^-\-^ mice (*n* = 4/genotype), **b**) *Bbs8*^*flox*^; *Cre-* and *Bbs8*^*flox*^; *Cre +* mice (n = 4/genotype, P value style APA: 0.12 non-significant, 0.033 (*), 0.002(**) and p < .001(***))

Next, we examined if reactive astrocytes had characteristic phenotypes of A1 and A2 type astrocytes. We then examined several gene expressions that have been reported to be inducers of A1 type astrocytes. We observed significantly increased expression of *Ligp1* (*p* < 0.005) and *Gbp2* (*p* < 0.002) in *Bbs8*^*−/−*^ mice, but expression of *Amigo2* (*p* > 0.05), *Fbin5* (*p* > 0.05), *Ht-t23* (*p* > 0.05), *Fkbp5* (*p* > 0.05), and *Psmb8* (*p* > 0.05) were unaltered (Fig. 8a), suggesting that some A1-like reactive astrocytes may be present. Next, we examined molecular inducers of A2 astrocytes and we observe some of the molecular markers of A2-subtype astrocytes are altered in *Bbs8*^*−/−*^ mice. Expression of *S10010a (p < 0.001)* and *Tm4sf1 (p < 0.007)* are significantly increased and expression of *Cd14 (p < 0.001)* is significantly reduced compared to WT littermates. Expression of *Sphk1* (*p* > 0.05) and *Cd109* (p > 0.05) are unchanged (Fig. 9a). These results indicate the presence of A2-subtype-like astrocytes in *Bbs8*^*−/−*^ mice. Together, the expression data suggests that both A1-like neurotoxic and A2-like neuroprotective astrocytes are present in congenital BBS8 knockout mice. However, neither subclass of astrocytes in the *Bbs8*^*−/−*^ mouse model is fully congruent with the established criteria for defining A1 and A2 reactive astrocytes [9].

**Fig. 10:**
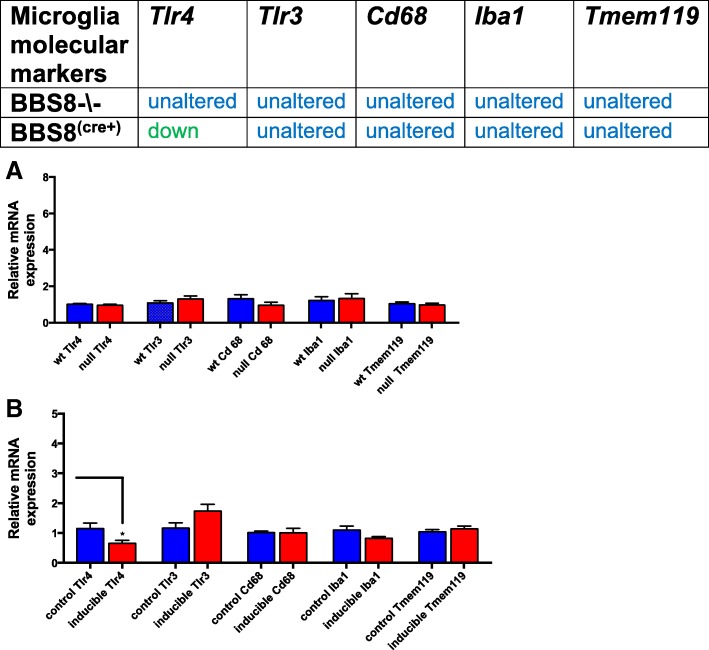
Loss or reduced BBS8 protein levels is not associated with microglia activation in brains of BBS8 mice Expression of molecular inducers of microglia in brains of control mice, Cre+, and *Bbs8*^*−/−*^ mice are summarized and graphed showing relative mRNA expression of molecular inducers of microglia activation in brains of control mice, Cre+, and *Bbs8*^*−/−*^ mice. Data are presented as Mean + SEM. **a** wt and BBS8^-\-^ mice (n = 4/genotype, **b**) *Bbs8*^*flox*^; *Cre-* and *Bbs8*^*flox*^; *Cre +* mice (n = 4/genotype, *P* value style APA: 0.12 non-significant, 0.033 (*), 0.002(**) and p < .001(***))

Since reactive astrocytes are often associated with reactive microglia we then assessed whether microglia were activated in *Bbs8*^*−/−*^ mice. We find expressions of *Iba1*(p > 0.05), *Cd68* (p > 0.05), *Tlr3* (p > 0.05), *Tlr4* (p > 0.05)*,* and *Tmem119* are unaltered (p > 0.05) (Fig. 10a). *Cd68* is an accepted molecular marker of reactive microglia and *Tmem119 is a specific marker for microglia and their* expression are not significantly altered in BBS8^-\-^ mice when compared to their wildtype littermates (Fig. 10a and Fig. 14a), Furthermore, TLR3 and TLR4 that are expressed on microglia and are activated in reactive microglia are not significantly altered in BBS8^-\-^ mice when compared to their wild type littermates. In addition, double immunofluorescence co-localization images show that pro-inflammatory cytokine IL1β does not co-localize to microglia cells (Fig. 13c). Thus, these results suggest that microglia are not activated in this mouse model at this age time point despite the presence of several molecular inducers of reactive astrocytes being significantly altered.

**Fig. 11:**
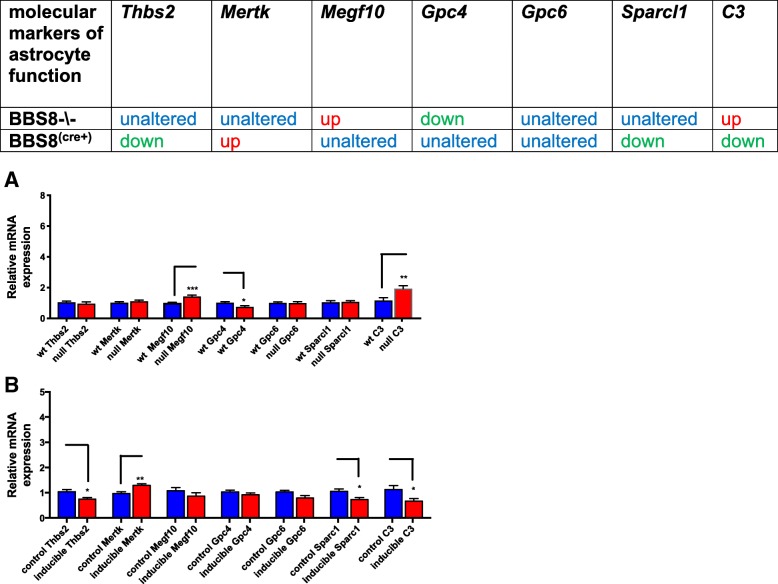
Loss of or reduced BBS8 protein levels are associated with dysregulated molecular inducers of astrocyte function in brains of BBS8 mice. Expression of molecular inducers of astrocyte function in brains of control mice, Cre+, and *Bbs8*^*−/−*^ mice are summarized and graphed showing relative mRNA expression of molecular inducers of astrocyte function in brains of control mice, Cre+, and *Bbs8*^*−/−*^ mice. Data are presented as Mean + SEM, **a**) wt and BBS8^-\-^ mice (n = 4/genotype), **b**) *Bbs8*^*flox*^; *Cre-* and *Bbs8*^*flox*^; *Cre +* mice (n = 4/genotype, P value style APA: 0.12 non-significant, 0.033 (*), 0.002(**) and *p* < .001(***))

The molecular components of phagocytic signaling pathways in healthy astrocytes are highly regulated. Synaptic phagocytosis is mediated through receptors on the astrocyte, and astrocytes assist in the formation of excitatory synapses by secreting thrombospondins and glypicans. Both of these processes are compromised in A1 astrocytes [9]. Since we observed A1-like astrocytes in *Bbs8*^*−/−*^ mice, we therefore examined the expression levels of molecular markers associated with astrocyte phagocytic functions and excitatory synapse formation in our mouse models. In *Bbs8*^*−/−*^ mice, expression of *C3* (*p* < 0.009) and phagocytic receptor *Megf10* (*p* < 0.001) are significantly increased while glypican *Gpc4* is significantly reduced (*p* < 0.014). Increased *C3* expression indicates the presence of A1-like astrocytes as upregulation of *C3* is seen in A1 astrocytes [9]. The mRNA levels of thrombospodin 2 *Thbs2* (*p* > 0.05), *Mertk* (p > 0.05), *Gpc6* (p > 0.05), and *Sparc1*(p > 0.05) are unchanged (Fig. 11a). These results indicate alterations in the phagocytic signaling capabilities of astrocytes in *Bbs8*^*−/−*^ mice.

**Fig. 12:**
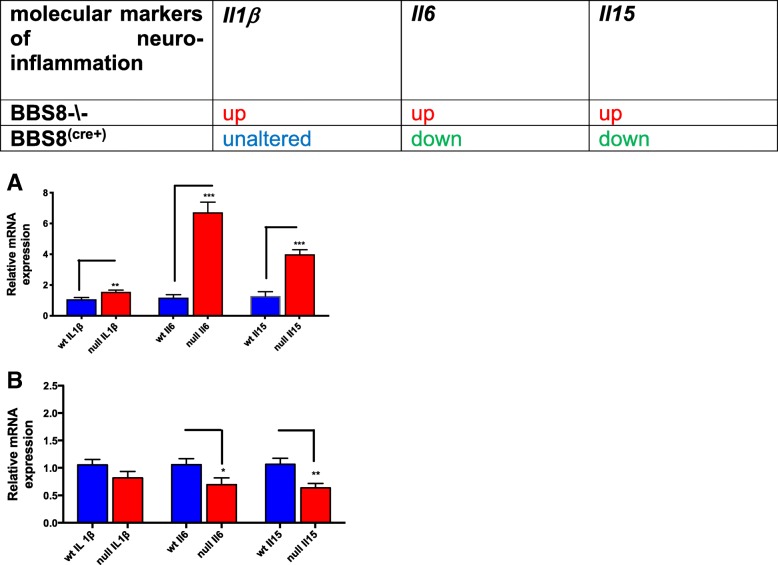
Loss of BBS8 protein is associated with increased expression of molecular inducers of neuro-inflammation in brains of BBS8^-\-^ mice. Expression of molecular inducers of neuro-inflammation in brains of control mice, Cre+, and *Bbs8*^*−/−*^ mice are summarized and graph showing relative mRNA expression of molecular inducers of neuro-inflammation in brains of corresponding control mice, *Bbs8*^*flox*^; *Cre +* mice, and *Bbs8*^*−/−*^ mice. Data are presented as Mean + SEM, **a**) wt and BBS8^-\-^ mice (n = 4/genotype), **b**) *Bbs8*^*flox*^; *Cre-* and *Bbs8*^*flox*^; *Cre +* mice (n = 4/genotype, P value style APA: 0.12 non-significant, 0.033 (*), 0.002(**) and p < .001(***))

**Fig. 13:**
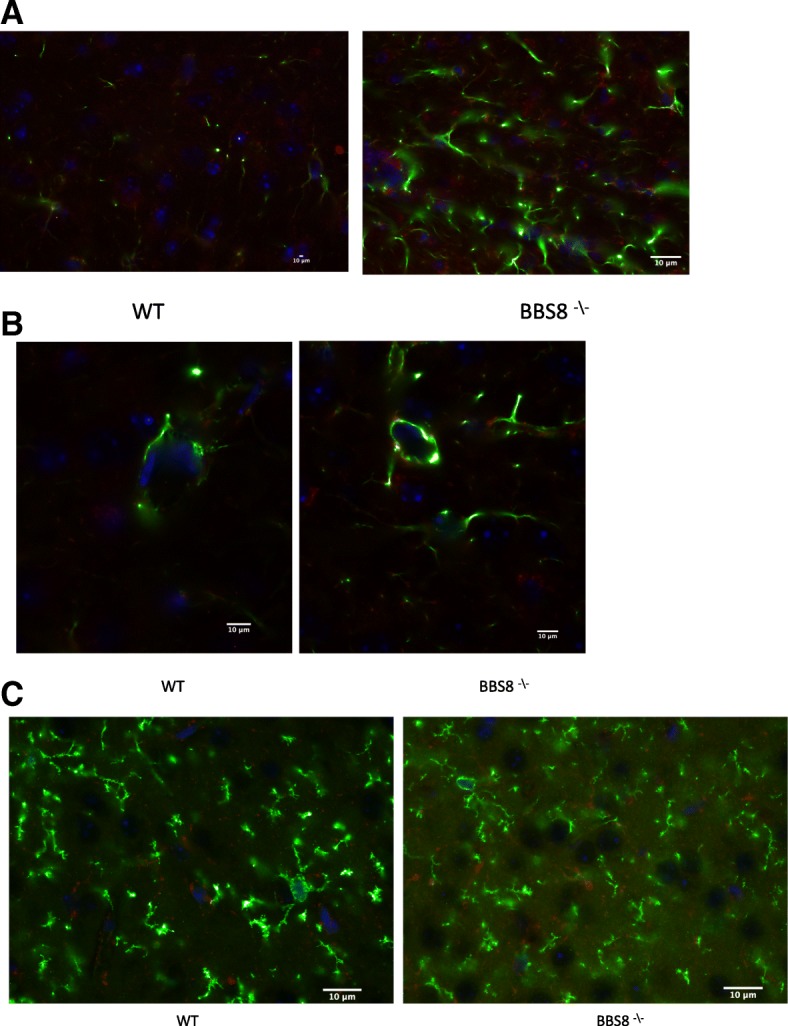
Increased pro-inflammatory cytokine IL1β co-localizes to reactive astrocytes in the corpus callosum of BBS8^-\-^ mice. **a** & **b,** Double immunofluorescence of pro-inflammatory IL1β (red) seen intermingled and co-localized to astrocytes (green) in corpus callosum of WT and BBS8^-\-^ mice at 40X and 63X magnification, **C)** Double immunofluorescence at 40X magnification of TMEM119 microglia cells (green) and pro-inflammatory Il1β (red) in cortex of WT and BBS8^-\-^ mice

Both reactive astrocytes and microglia secrete pro-inflammatory cytokines [9, 32], and specific pro-inflammatory cytokines secreted by microglia induce reactive A1 subtype astrocytes [9]. Therefore, we analyzed gene expression of pro-inflammatory cytokines that have been associated with reactive astrocytes and neuro-inflammation [9]. Expression of the pro-inflammatory markers *ll1β*, (*p* < 0.006), *Il6* (p < 0.001), and *Il15* (p < 0.001) are significantly increased in *Bbs8*^*−/−*^ mice when compared to wild type littermates (Fig. 12a), which indicate the presence of neuroinflammation in this mouse model. We also examined if pro-inflammatory cytokines IL1β co-localizes to reactive astrocytes and find increased IL1β immunoreactivity in corpus callosum of BBS8^-\-^ mice when compared to WT mice and that IL1β co-localizes to reactive astrocytes and not to microglia cells in BBS^-\-^ mice (Fig. 13a and b).

**Fig. 14:**
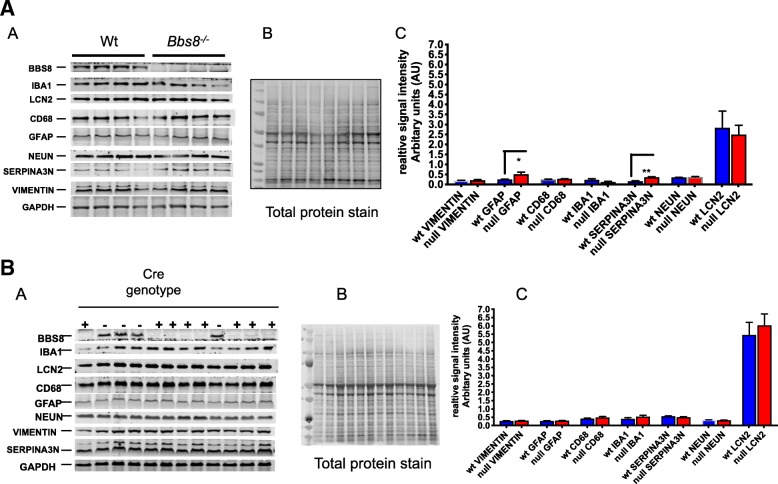
Loss of or reduced BBS8 protein levels are associated with increased protein levels of reactive astrocyte markers without alteration of protein markers of microglia activation in brains of BBS8^-\-^ mice. Relative levels of protein changes are summarized, and figure shows steady state protein levels on the left, in the center image of a representative total protein stain, and on the right graph of western blot analysis from total brain protein lysate of corresponding control mice, *Bbs8*^*flox*^; *Cre+*, and *Bbs8*^*−/−*^ mice. Data are presented in the graph as relative mean signal intensity (arbitrary units (AU)) + SEM. **a** wt and BBS8^-\-^ mice (n = 4/genotype, *p < 0.04), **p < 0.002), **b)** *Bbs8*^*flox*^; *Cre-* and *Bbs8*^*flox*^; *Cre +* mice (− = *Bbs8*^*flox*^; *Cre-* and + = *Bbs8*^*flox*^; *Cre +* (*n* > 4/genotype, *p* > 0.05)

Western blot analyses on brain lysates of *Bbs8*^*−/−*^ mice show that relative protein levels of GFAP (*p* < 0.04) and SERPNA3N (*p* < 0.002) are significantly increased while protein levels of VIMENTIN (*p* > 0.05), LCN2 (p > 0.05), IBA1 (p > 0.05), CD68 (p > 0.05), and NEUN (p > 0.05) are unaltered compared to WT littermate mice (Fig. 14a).

**Fig. 15:**
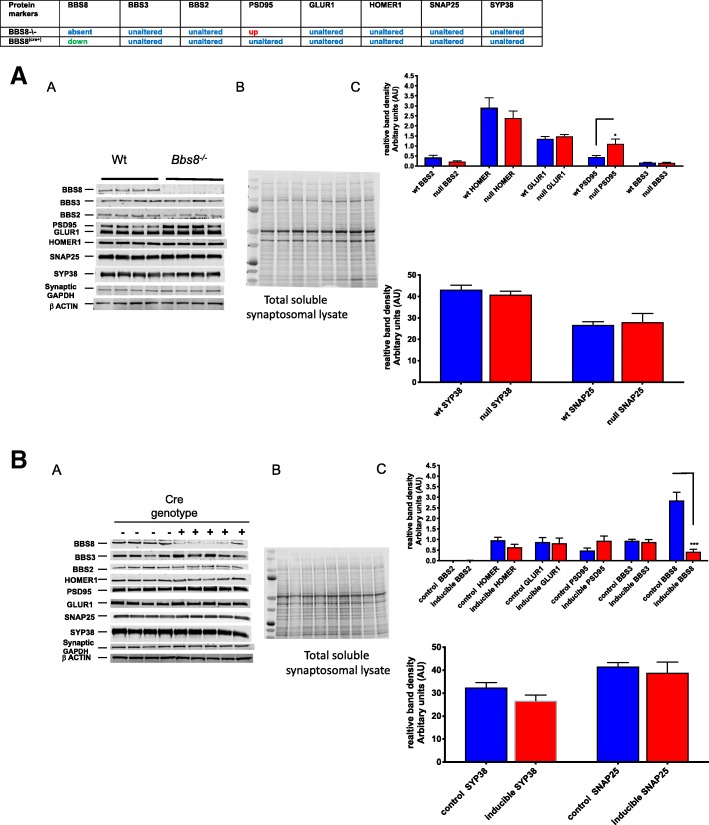
Loss of or reduced BBS8 protein levels are associated with increased protein levels of specific postsynaptic density proteins in brains of BBS8 mice. Relative levels of protein changes are summarized and steady state protein levels are shown on the left, in the center a representative image of total protein stain, and on the right graph showing quantitative analysis from western blots of corresponding control mice, *Bbs8*^*flox*^; *Cre+*, and *Bbs8*^*−/−*^ mice total brain synaptosome lysates. Data are presented in the graph as relative mean signal intensity +SEM. **a**) wt and BBS8^-\-^ mice (n = 4/genotype, *p < 0.043), **b**) *Bbs8*^*flox*^; *Cre-* and *Bbs8*^*flox*^; *Cre +* mice (n > 4/genotype ***p < 0.0004)

Synaptic function and number are often negatively affected when astrocytes become reactive [9]. To assess if reactive astrocytes in *Bbs8*^*−/−*^ mice affected synaptic protein levels of synapses, we next examined if pre- and post-synaptic protein levels in synaptosomes containing isolated nerve terminals, were altered in *Bbs8*^*−/−*^ mice. We observed significantly increased levels of PSD95 (p < 0.04) in *Bbs8*^*−/−*^ mice compared to controls via Western blot, while protein levels of GLUR1 (p > 0.05), SNAP25 (p > 0.05), SYP(p > 0.05), and HOMER1 (p > 0.05),) remain unaltered (Fig. 15a). Since PSD95 functions as a scaffold to assemble a variety of signaling molecules around NMDAR, these data suggest synaptic NMDAR dysfunction in *Bbs8*^*−/−*^ mice.

### Postnatal reduction of BBS8 in an inducible BBS8 mouse model is associated with reactive astrocytes and reduced pro-inflammatory *Il6* transcripts, independent of microglia activation

In order to determine which phenotypes observed in the BBS8 congenital knockout were due to maintenance functions of the BBSome and which were likely caused by either developmental roles or hydrocephalus, we characterized *Bbs8*^*flox*^; *Cre +* mice, an inducible knockout model of BBS8, in a similar manner to that described for the congenital knockout. *Bbs8*^*flox*^; *Cre +* mice and *Bbs8*^*flox*^; Cre- littermates were treated with tamoxifen at P9, P12, and P15 to induce deletion of *Bbs8* in Cre + mice [29].

We performed gene expression analysis on total brain RNA from *Bbs8*^*flox*^; *Cre +* and *Bbs8*^*flox*^; Cre- littermates (*n* = 4 for each genotype). We examined if pan-reactive, A1- and A2- molecular inducers were altered when BBS8 was deleted postnatally. We observed significantly increased expression of *Gfap* (*p* < 0.004) and *Serpina3n* (*p* < 0.043) and significantly reduced expression of *Lcn2* (*p* < 0.008) in *Bbs8*^*flox*^; Cre + mice, while expression of *Steap4* (*p* > 0.05), *Vimentin* (p > 0.05), and *Aspg* (*p* > 0.05) were unchanged when compared to their control litter mate mice (Fig. 7b). These data indicate reactive astrocytes are present and are consistent with the observed increased astrocyte immunoreactivity in *Bbs8*^*flox*^; *Cre +* mice.

We then tested for the presence of A1 and A2 reactive astrocytes. In *Bbs8*^*flox*^; *Cre +* mice, expression of *H2-t23* (*p* < 0.01) and *Gbp2* (p < 0.04) are significantly reduced compared to controls. *Amigo2* (*p* > 0.05), *Ligp1* (p > 0.05), *Fkbp5* (p > 0.05), *Psmb8* (p > 0.05), and *Fibin5* (*p* > 0.05) are unaltered (Fig. 8b). Additionally, expression of *Sphk1* (*p* < 0.006) is significantly reduced and expression of *Cd109* (*p* > 0.05), *Cd14* (p > 0.05*)*, *S10010a* (p > 0.05), and *Tm4sf1* (p > 0.05) are unaltered in *Bbs8*^*flox*^; *Cre +* mice (Fig. 9b). Thus, at this time point, it is probable that neither activated A1-like nor A2-like subtype reactive astrocytes are present in *Bbs8*^*flox*^; *Cre +* mice, despite the upregulation of pan-reactive astrocyte markers.

We then examined whether molecular inducers of astrocyte function are altered in inducible BBS8 mice. When compared to their *Bbs8*^*flox*^; Cre- littermates, expression of the phagocytosis receptor *Mertk* (*p* < 0.003) is significantly increased in *Bbs8*^*flox*^; *Cre +* mice, while expression of *Thbs2* (*p* < 0.013), *C3* (*p* < 0.027), and *Sparcl1* (p < 0.02) are significantly reduced (Fig. 11b). The expression levels of glypicans *Gpc4* (*p* > 0.05) and *Gpc6* (p > 0.05) and *Megf10* (p > 0.05) are unchanged. These results suggest there is increased transduced phagocytosis signaling and that excitatory synapse formation is compromised in *Bbs8*^*flox*^; *Cre +* mice through a different molecular pathway than observed in the congenital knockout.

We next examined molecular inducers of reactive astrocytes and find that in *Bbs8*^*flox*^; *Cre +* mice, expression of *Tlr4* (*p* < 0.03) is significantly reduced, but *Iba1* (p > 0.05), *Cd68* (p > 0.05), *Tlr3* (*p* > 0.05) and *Tmem119* (*p* > 0.05) remain unchanged (Fig. 10b). The accepted molecular marker of reactive microglia *Cd68* is not significantly altered at the mRNA and protein level (Figs. 10b and 14b). These data suggest that at 1 month of age, reactive astrocytes are present without microglial activation in inducible BBS8 knockout mice; this phenotype is similar to that of *Bbs8*^*−/−*^ mice. However, we were unable to detect evidence of altered neuroinflammation markers in *Bbs8*^*flox*^; *Cre +* mice. Expression of *Il6* (*p* < 0.04) and *Il15* (*p* < 0.005) are significantly reduced and expression of *Il1β* (p > 0.05) is unaltered (Fig. 12b), identifying yet another difference between congenital and postnatal deletion of BBS8.

Together, the mRNA expression data suggest that in *Bbs8*^*flox*^; *Cre +* mice molecular markers of pan reactive astrocytes are increased, and some molecular markers associated with astrocyte function are dysregulated. Unlike the congenital knockout of BBS8, A1- and A2-like astrocyte molecular markers and neuroinflammation are not observed in the inducible BBS8 knockout mice. Thus, the molecular pathways leading to astrocyte reactivity in congenital and inducible BBS8 mouse models appear to be molecularly distinct.

*Bbs8*^*flox*^; *Cre +* mice had significantly reduced levels of BBS8 (*p* < 0.001) compared to *Bbs8*^*flox*^; Cre- mice on western blot, demonstrating efficient tamoxifen-induced deletion of the gene. Protein levels of BBS2 (p > 0.05) and BBS3 (*p* > 0.05) are unaltered in *Bbs8*^*flox*^; *Cre +* mice, supporting the specificity of excision (Fig. 14b).

Unlike congenital knockouts, *Bbs8*^*flox*^; *Cre +* mice do not display differences from their control littermates in levels of GFAP, SERPNA3N, VIMENTIN, LCN2, IBA1, CD68, and NEUN in brain lysates, despite changes in transcript levels. We also observed no differences in the synaptosome proteins PSD95, HOMER1, SNAP25, SYP, and GLUR1 (Fig. 15a and b). These results suggest that synaptic signaling is not compromised in inducible, postnatal knockouts of BBS8, and at this age post-transcriptional modulation may be occurring in this mouse model.

## Discussion

### Loss of BBSome function leads to astrocyte reactivity independent of hydrocephalus

Primary cilia regulate development of the CNS by sensing local environmental signals and promoting neuronal proliferation and differentiation [33–35]. Due to this critical role in the brain, ciliopathies often present with cognitive impairment and cortical malformations [23–25]. Primary cilia are also present on astrocytes [36]. Astrocytes become reactive in response to a specific insult, and they become hypertrophic and overexpress intermediate filament GFAP at the protein level [37, 38]. In addition, they may show morphological and functional changes [7, 9, 39–41].

We observed increased GFAP and VIMENTIN immunoreactivity in multiple different congenital knockout mouse models of BBS prior to the development of obesity, suggesting that loss of BBSome function causes reactive astrocytes. However, since congenital knockouts of BBS develop hydrocephalus before birth, these models do not allow us to distinguish between loss of BBSome function and hydrocephalus as the cause for reactive astrocytes. Since early postnatal deletion of *Bbs8* results in loss of BBSome function [29] but does not cause hydrocephalus at one month of age, we were able to use this model to identify the primary source of reactive astrocytes in BBS mouse models. The presence of reactive astrocytes in *Bbs8*^*flox*^; *Cre +* mice indicates that loss of BBSome function is sufficient to result in reactive astrocytes, even in the absence of hydrocephalus.

In *Bbs8*^*−/−*^ mice, we identified significantly increased levels of *Gfap* mRNA expression that correlated with increased protein levels. However, significantly increased expression of *Lcn2* did not result in increased LCN2 protein levels in *Bbs8*^*−/−*^ mice. A discrepancy is also observed for *Serpina3n*: mRNA expression is significantly reduced, but the protein level of SERPINA3N is significantly increased in *Bbs8*^*−/−*^ mice (Fig. 8a). These data suggest that post-transcriptional control regulates the level of pan-reactive astrocyte proteins in *Bbs8*^*−/−*^ mice.

### Loss of BBSome function does not result in microglia activation, despite astrocyte reactivity

Increased GFAP expression and altered morphology are usually associated with reactive microglia and neuro-inflammation. Generally, the innate immune system is responsible for detecting and clearing surplus neurotransmitters, invading pathogens, and aged glycated proteins to maintain a healthy environment for neuronal and glial cells [42]. However, we observe no increase in IBA1 immunostaining in either of our BBS8 mouse models. These results were confirmed by assessing the mRNA expression levels of molecular inducers of reactive microglia *Iba1*, *Tmem119, and Cd68* (Fig. 10a) and protein levels of IBA1 and CD68 in brain lysates (Fig. 14a). Expression of *Iba1*, *Cd68*, and *Tmem119* transcripts as well as IBA1 and CD68 protein levels are unchanged which is consistent with observed immunohistochemistry data. We also examined expression changes in Toll like receptors 3 and 4 as they are expressed on microglia and are activated in reactive microglia [9, 43, 44]. We do not observe changes in expression of *Tlr3* and *Tlr4* in brains of *Bbs8*^*−/−*^ mice. Together, these findings indicate that microglia are not activated in brains of *Bbs8*^*−/−*^ mice at 1 month of age. We observed similar results in *Bbs8*^*flox*^; *Cre +* mice, demonstrating that loss of BBSome function does not induce microglia activation at 1 month of age (Fig. 10b and Fig. 13b). The fact that the accepted molecular marker of reactive microglia CD68 showing no significant changes at both mRNA level and protein levels (Fig. 10b and Fig. 14b,), observed ramified morphology with thin branched process from microglia cells (Figs. 5, 13c), no significant changes in expression of microglia specific molecular marker TMEM119, no significant changes in TLR3 and TLR4 expressions, and pro-inflammatory cytokine IL1β does not co-localize to microglia cells but rather to astrocytes suggest there is no evidence of reactive microglia at this age in this disease model. It is possible that there may be regional differences in reactive astrocytes and microglia molecular phenotypes. However, examining very closely across different brain regions under the microscope, we did not observe presence of ameboid microglia morphology. We consistently observed ramified microglia morphology with thin branched process from the microglia cells as shown in Figs. 5 and 13c. This is supported from results obtained from mRNA and protein levels of cd68 which is the standard molecular marker of reactive microglia. Therefore, we conclude that reactive astrocytes and neuroinflammation are present in the absence of reactive microglia. We propose that reactive astrocytes are of novel type in this disease model as it could be due to defective cilia signaling in either astrocyte, neuron cells or other CNS cells signaling “help me” to astrocytes that may give rise to reactive astrocyte phenotype. Therefore, these reactive astrocytes may represent novel type of reactive astrocytes in this disease context as they don’t confer to fully to neither A1 nor A2 type of reactive astrocytes.

Thus, our results indicate that reactive astrocytes are induced in the absence of microglia activity, potentially pointing towards a novel mechanism of astrocyte activation caused by compromised function of primary cilia.

### Developmental and postnatal roles of the BBSome in brain health and astrocyte reactivity

We observed multiple differences between the BB8 congenital knockout model (*Bbs8*^*−/−*^) and the Bbs8 inducible deletion model (*Bs8*^*flox*^; *Cre+*). We hypothesize that these differences arise from the congenital nature of the defect in *Bbs8*^*−/−*^ mice, and/or may be associated with the hydrocephalus. While we detect increased GFAP mRNA levels in both BBS8 congenital and induced deletion mutant mice we observe no differences in GFAP protein levels by Western blot analysis in *Bbs8*^*flox*^; *Cre +* mice, indicating that loss of BBSome function is not solely responsible for the increase in astrocyte reactivity observed in the congenital knockout models of BBS. Additionally, the two BBS8 mutant models display molecularly distinct expression patterns of genes associated with pan-reactive, A1-subtype, and A2-subtype astrocytes. This suggests that the reactive astrocytes in the BBS8 congenital knockout are molecularly distinct from those in the inducible knockout, and that postnatal loss of BBSome function causes astrocyte reactivity differently than congenital loss of BBSome function.

While we observed pan-reactive astrocytes in both models of BBS8 loss, only the congenital knockout had a transcriptional profile consistent with the presence of A1-like neurotoxic and A2-like neuroprotective astrocytes. The characterization of astrocytes in *Bbs8*^*flox*^; *Cre +* mice will require further study, including aging of the mice to determine whether astrocytes will eventually develop into A1/A2 subtypes in this model.

Pro-inflammatory cytokines have been reported to be secreted by reactive astrocytes and microglia [32, 45–47]. We examined whether the reactive astrocytes in *Bbs8*^*−/−*^ mice are associated with the pro-inflammatory cytokines *Il6*, *Il1β*, and *Il15*, which have been implicated in neuro-inflammation. We observe significantly increased levels of *Il6 (p < 0.001)*, *Il1β* (*p* < 0.005) and *Il15 (p < 0.001)* in *Bbs8*^*−/−*^ mice (Fig. 12a), and pro-inflammatory cytokine 1L1β co-localizes to reactive astrocytes in BBS8^-\-^ mice (Fig. 13a and b).Thus, reactive astrocytes are associated with increased pro-inflammatory cytokines in *Bbs8*^*−/−*^ mice*.* We also attempted to co-localize IL6 and IL15 cytokines in microglia and astrocytes but unfortunately these antibodies did not work in our hands. Therefore, it’s difficult to conclude the source of Il6 and Il15 cytokines. However, on the basis of no changes in CD68, and IBA1 mRNA and protein levels and co-localization images suggest that reactive microglia are not present and that pro-inflammatory cytokines that are significantly increased may be secreted by astrocytes or other CNS cells*.* Conversely, in *Bbs8*^*flox*^; *Cre +* mice, mRNA expression of the pro-inflammatory cytokines *Il6 (p < 0.04)*, and *Il15 (p < 0.004)* is significantly decreased, suggesting that reactive astrocytes do not induce pro-inflammatory cytokines in this mouse model at this time point. However, this does not rule out the possibility that with sustained astrocyte reactivity, significantly increased pro-inflammatory cytokines at a later time point in *Bbs8*^*flox*^; *Cre +* mice may be found.

Since A1-like astrocytes and pro-inflammatory cytokines are neurotoxic [48], we examined NEUN protein levels in brain lysates and immunoreactivity in brain slices of *Bbs8*^*−/−*^ mice and their control littermates. NEUN protein levels in *Bbs8*^*−/−*^ mice were not different then their littermates by either Western blotting or immunohistochemistry, suggesting that there is no significant mature neuronal cell loss despite increased expression of pro-inflammatory markers in *Bbs8*^-\-^ mice at 1 month of age (Fig. 6d and 12a). However, further studies on a time course examining changes of markers of reactive astrocytes, microglia, and pro-inflammatory cytokine in relation to neuronal cell loss from sub-brain regions are required for an in depth understanding of the type of reactive astrocytes and their influence on brain health in *Bbs8*^*−/−*^ mice.

We cannot rule out the inefficiency of tamoxifen excision as a factor in the differences between the congenital and inducible knockout mice. Postnatal deletion at this time point is efficient enough to cause both retinal degeneration [29] and late onset obesity (J. Garrison, unpublished data), but it is possible that the deletion threshold for development of brain phenotypes is higher than that of retinal phenotypes. Despite this, the differences between congenital and inducible knockout mice of BBS8 support the hypothesis that reactive astrocytes are highly heterogeneous as well as disease and context specific.

### Significantly increased protein levels of PSD95 suggest dysregulated synaptic function and compromised neuronal signaling due to congenital loss of BBSome function

Synaptic functions are modulated by numerous proteins in the synaptic junction. Post synaptic density 95 (PSD95) promotes maturation of synapses and strongly influences synaptic strength and plasticity [49, 50]. Increased PSD95 potentiates AMPAR-mediated excitatory postsynaptic currents by driving GluR1 into synapses [51, 52]. We find that PSD95 protein levels in brain synaptosomal lysates are significantly increased (*p* < 0.04) and other synaptosomal proteins are unaltered in *Bbs8*^*−/−*^ mice when compared to their wild type littermates (Fig. 15a).

We observe no such defect in *Bbs8*^*flox*^; *Cre +* mice, suggesting that this phenotype may occur in development. However, we did detect increased expression of the astrocyte phagocytosis receptor *Mertk* in *Bbs8*^*flox*^; *Cre +* mice, and expression of *Thbs2, Sparc1,* and *C3* are significantly reduced. Together, these data suggest that reactive astrocyte influence synaptic formation which may be compromised in *Bbs8*^*flox*^; *Cre +* mice, despite no discernable difference in PSD95.

Increased PSD95 could arise due to post-translational modification or mis-trafficking of PSD95, as BBS proteins are known to play a role in trafficking. When examined if other BBS proteins besides BBS8 were present in the synaptic lysates. New findings of BBS2 and BBS3 proteins in synaptic lysates suggests that there may be a role for BBS proteins at the synaptic junction. Dysregulation of BBSome function could lead to neurons sending a distress signal which in turn lead to the development of reactive astrocytes [53] or that dysregulated BBSome function within astrocytes could lead to astrocyte reactivity. An inability of the congenital knockout mice to upregulate the A2-subtype neuroprotective reactive astrocytes over the neurotoxic A1-subtype could lead to further astrocyte reactivity, increased pro-inflammatory cytokines, and loss of synaptic function.

Importantly, no indications of neuronal cell loss, as assayed by NeuN staining and western blot analyses, that were detected in both BBS8 mouse model suggests that the BBSome is not required for neuronal viability, and that neuronal death is unlikely to be contributing to reactive astrocytes. It is possible that the observed astrocyte reactivity could be a cell-autonomous phenotype, and not dependent on neuronal or microglial dysfunction.

Further work will be necessary to characterize, in-depth, the astrocytes in congenital knockouts and inducible deletion models of BBS8. Our in vivo findings show that when BBS8 protein levels are dysregulated, reactive astrocytes are present. These reactive astrocytes have distinct molecular characteristic phenotypes that depend on the timing of loss of BBSome function, adding further support that reactive astrocytes are highly heterogeneous, disease, and context specific.

Our study shows that molecularly distinct reactive astrocytes are present in the absence of BBSome function in two different mouse models of BBS8 dysregulation. Congenital loss of BBSome function leads to increased molecular inducers of Pan-reactive, A1- and A2-like astrocytes, altered molecular inducers of astrocyte function, and presence of neuroinflammation in the absence of microglia activation. Inducible, postnatal loss of BBSome function leads to increased molecular inducers of Pan-reactive *Gfap*, altered molecular inducers of astrocyte function in the absence of microglia activation. Our results further corroborate the heterogenous nature of reactive astrocytes.

Significantly increased GFAP and VIMENTIN immunoreactivity in SVZ suggests that different brain regions needs to be further investigated for molecular phenotyping of each brain region to address if there is a unifying molecular signature that can be assigned to their morphology, gene expression, and functional differences.

Astrocytes are the most abundant cells of the central nervous system, and these cells provide nutrients and recycle neurotransmitters and play a crucial role in brain neuro-inflammatory response. Therefore, further characterization of reactive astrocytes in vivo as opposed to in vitro will facilitate not only a better understanding of types of reactive astrocytes present in the brain but also the development of therapeutics for injured and diseased brain for a wide variety of disorders [54].

## Abbreviations

BBS: Bardet-Biedl Syndrome
CNS: Central nervous system
GFAP: Glial Fibrillary Acidic Protein
PSD95: Post synaptic density 95
